# Synergistic Combination of Electrolysis and Electroporation for Tissue Ablation

**DOI:** 10.1371/journal.pone.0148317

**Published:** 2016-02-11

**Authors:** Michael K. Stehling, Enric Guenther, Paul Mikus, Nina Klein, Liel Rubinsky, Boris Rubinsky

**Affiliations:** 1 Inter Science GmbH, Biophysics, Luzern, Switzerland; 2 Institut fuer Bildgebende Diagnostik - Tumortherapy Center, R&D, Offenbach, Germany; National Research Council, ITALY

## Abstract

Electrolysis, electrochemotherapy with reversible electroporation, nanosecond pulsed electric fields and irreversible electroporation are valuable non-thermal electricity based tissue ablation technologies. This paper reports results from the first large animal study of a new non-thermal tissue ablation technology that employs “Synergistic electrolysis and electroporation” (SEE). The goal of this pre-clinical study is to expand on earlier studies with small animals and use the pig liver to establish SEE treatment parameters of clinical utility. We examined two SEE methods. One of the methods employs multiple electrochemotherapy-type reversible electroporation magnitude pulses, designed in such a way that the charge delivered during the electroporation pulses generates the electrolytic products. The second SEE method combines the delivery of a small number of electrochemotherapy magnitude electroporation pulses with a low voltage electrolysis generating DC current in three different ways. We show that both methods can produce lesion with dimensions of clinical utility, without the need to inject drugs as in electrochemotherapy, faster than with conventional electrolysis and with lower electric fields than irreversible electroporation and nanosecond pulsed ablation.

## Introduction

Minimally invasive surgery employs various tissue ablation technologies, each with their own advantages, disadvantages and specific use. Electric currents passing through a biological medium produce a number of biophysical and biochemical effects which are used in tissue ablation. This study deals with the use of a combination of two different electricity driven phenomena, electrolysis and electroporation.

The electrochemical reactions known as electrolysis occur at the surface of electrodes submerged in an ionic conducting media, during the passage of an electric current [1]. New chemical species are generated at the interface of the electrodes as a result of electron transfer between the electrodes and the ions in solution. The new chemical species diffuse away from the electrodes, into tissue, in a process driven by electrochemical potentials. In physiological solutions, electrolytic reactions yield changes in pH, resulting in an acidic region near the anode and a basic region near the cathode. The cytotoxic environment developing due to local changes in pH, as well as the presence of some of the new chemical species formed during electrolysis of the solution, cause cell death. Electrolysis is harnessed for tissue ablation in medicine, since the early 1800’s [2]. The field has experienced a revival in the mid 1970’s, with the work of Nordenstrom [3,4]. During the last two decades, substantial research was done on tissue ablation by electrolysis [5–24]. The cited studies include cell and animal experiments, mathematical modeling and clinical work. From an operational standpoint, electrolysis requires very low voltages and currents, providing advantages relative to other ablation techniques, e.g. reduced instrumentation complexity. It is, however, a lengthy procedure, controlled by the process of diffusion and the need for high concentrations of electrolytically-produced ablative chemical species.

Permeabilization of the cell membrane through the application of very brief, high-magnitude electric field pulses characterizes the bioelectric phenomenon of electroporation [25–34]. The effect on the cell membrane is a function of the electric field strength and pulse time length [35–37]. Lower electric fields produce reversible pores in the lipid bilayer, allowing the introduction of molecules such as genes and drugs into cells [32,38]. Higher electric fields produce irreversible defects (pores), resulting in a cell membrane that does not reseal after the field is removed [35]. Reversible and irreversible electroporation have numerous medical applications [39]. Reversible electroporation techniques have been combined with anticancer drugs such as bleomycin to target cancerous tissues for successful clinical use in the field of electrochemotherapy [40]. Reversible electroporation for electrochemotherapy employs voltage over distance between electrodes in the range of between 300 kV/cm and 1–1.5 kV/cm and usually eight pulses [40]. The use of non-thermal irreversible electroporation (NTIRE) for tissue ablation is a more recent addition to the armamentarium of tissue ablation techniques available to surgeons [41–44]. Its use results in direct cell death without the need to introduce drugs or other molecules to facilitate the treatment process [45]. Irreversible electroporation usually employs up to one hundred pulses of microsecond length and electric fields in the single kV/cm range. Another recent non-thermal and non-chemical approach, known as nanosecond pulsed electric fields, uses much shorter pulses in the nanosecond domain, and increased electric field strengths in the range of tens or hundreds of kV/cm [46–48].

A major advantage of tissue ablation by electroporation is the relative speed of the procedure in comparison to any other ablation technique. Furthermore, because the procedure primarily affects the cell membrane, critical features of the extracellular matrix are spared, and this ablation modality can be used to treat tissues, such as the pancreas, without concern for collateral damage [45]. Current pulsed electric field tissue ablation approaches, however, have their respective disadvantages. Electrochemotherapy requires the injection of drugs into the tissue, while the high electric fields used in irreversible electroporation were found to cause muscle contractions, electric discharge associated with high pressure waves and may affect the electrical function of the heart.

Two recent studies, performed in a small animal model, have shown that combining electroporation with electrolysis has a synergistic effect and yields a tissue ablation technique that has certain advantages over tissue ablation with electroporation or electrolysis delivered separately [49,50] We will refer to the combination of electroporation and electrolysis “synergistic electroporation and electrolysis” (SEE). We have developed several methods of SEE. One of them employs electrolysis as the central tissue ablation modality and adds reversible electroporation electrochemotherapy-type pulses to permeabilize the cell membrane, making the cell more susceptible to lower amounts of products of electrolysis [50]. Several different combinations of electrolysis and electroporation are possible [50]. Another method of SEE is based on the fact that electric currents produce a variety of different effects, simultaneously [49] Electric currents which accompany the electroporation pulses also generate products of electrolysis. Therefore, we applied multiple reversible electroporation electrochemotherapy-type pulses to also generate products of electrolysis that are toxic and thus cause cell death to the reversible permeabilized cell membrane. This method of SEE ablation causes cell death with lower electric field pulses than the pulses used in irreversible electroporation or nanopulse ablation alone [49].

The study reported in was acute and done on a small animal model. While the principles of SEE were demonstrated in [49,50] the tissue ablation parameters and geometrical configurations used in those papers are not clinically applicable. Therefore, the primary goal of this pre-clinical study is to expand on the concepts introduced in [49,50] and explore the use of SEE in settings that are more relevant to clinical use. To this end we have tested a variety of potentially clinical SEE protocols. in an *in vivo* pig liver model. Our results confirmed the findings of [49,50] in a large animal model, and provide further clinical insight into the use of the combination of electrolysis and electroporation for tissue ablation with relevance to clinical procedures.

## Materials and Methods

The study was conducted on *in vivo* pig liver and approved by the PMI’s Institutional Animal Care and Use Committee IACUC (PMI—Pre Medical Inovation, San Carlos, CA, USDA number: 93-R-0506, Study number: ANS 2094). We used three female pigs, weight 30 kg to 40 kg, treated in accordance with Good Laboratory Practice regulations as set forth by the 21 Code of Federal Regulations (CFR) Part 58. Each procedure started with anesthetization of the animal under general anesthesia per SOP #33156. Preanesthetic medication was Telazol 4.0 mg/kg (2.0 ml) IM and Atropine 0.02 mg/kg (1.8 ml) IM. Anesthetic induction was done by Isoflurane with Oxygen, 2%/2L/minute via mask. Possible postoperative pain was ameliorated by Buprenorphine 0.01 mg/kg IM Pre-med at recovery and Carprofen 4 mg/kg at extubation/recovery. Antibiotics administered during surgery was Cefazolin 25 mg/kg IV every 2 hours. In addition, pancuronium (0.1 mg/kg, at a dose of 1 mg/ml) was administered through an IV prior to the procedure, to reduce muscle contractions during the application of the electrical pulses. Pancuronium (0.05 mg/ml at 1 mg/ml) was administered throughout the procedure as needed. The liver was exposed via a midline incision. The treatment was delivered using two 18 gauge Titanium needles (Inter Science GmbH, Ch) with a sliding insulating sheath inserted in the liver. We have used Ti needles to eliminate possible electrolytic products involving the electrode materials. The 18 gauge variable length electrodes were custom designed for the delivery of both electroporation and electrolytic pulse sequences.

**Fig 1:**
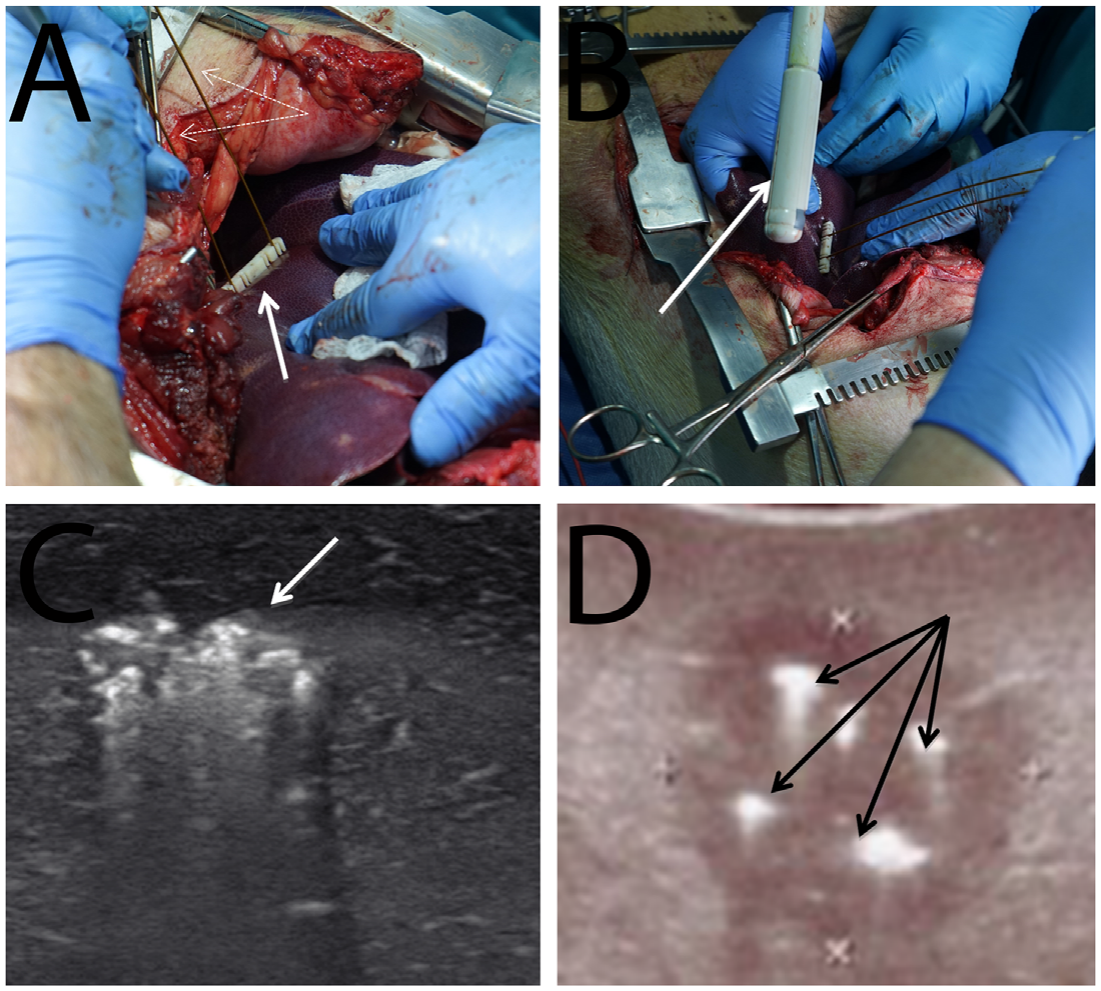
Photographs of the surgery and the ultrasound images taken during the procedure. (A) Placement of two electrodes (dashed arrows) through a grid (solid line arrow) into the liver. (B) Use of an intraoperative ultrasound (solid arrow) to monitor the placement of the probes and the tissue ablation. (C) Ultrasound images of an electrolytic treatment with two electrodes (note the bright hyperechoic area—marked by arrow). (D) Ultrasound images of an electroporation treatment with four electrodes (note the bright hyperechoic area—marked by arrows). (Bottom right figure used with permission of the publisher [44]).

Two electrodes were inserted in the liver in a roughly axial parallel configuration, normal to the liver surface, under ultrasound monitoring (Fig 1). Ultrasound images were also taken throughout the procedure. Square DC electroporation pulses were applied to the liver through the electrodes from a DC pulse generator (BTX, Harvard Instruments, USA). The electrodes were also connected to an Arbitrary Function Generator (AFG 3102, Tektronix, Beaverton, OR) to produce a constant current for a fixed period of time for electrolysis. The parameters varied in this study were: the voltage and the current of the electroporation and electrolysis pulses, the number of pulses, the distance between electrodes as well as the sequence of electrolysis and electroporation pulses. Animals were sacrificed at 24 hours. The pigs were euthanized using Euthasol 1 ml/lb IV.

To fix the liver in its current state for microscopic viewing, a Foley catheter was placed into the descending aorta and the hepatic vein was snipped off for drainage of the affluent. The liver was flushed with physiological saline for ten minutes at a hydrostatic pressure of 80 mmHg from a pressurized IV drip. Immediately following saline perfusion, a 10% formalin fixative was perfused in the same way for ten minutes. The liver lobe in which the SEE lesion was made was removed and stored in the same formalin solution. For microscopic analysis, the tissue was bread loafed perpendicular to the capsule surface and parallel to the needle tracts. All cassettes were processed routinely from 10% phosphate buffered formalin to wax blocks. Five micrometer sections were made from each block and stained with hematoxylin and eosin for histologic examination. The stained samples were scanned with a digital microscope D-Sight Fluo 2.0 (A. Menarini, Diagnostics, S.r.l, Firenze, Italy) in preparation for histological examination. The digitized histological images were examined blindly by an independent histology service company and reports were prepared (Narayan Raju, Inc). The focus of the histology was to verify the extent and nature of tissue ablation with SEE; in particular in relation to locations relative to the electrodes and the continuity of the lesion between the electrodes.

To compare lesion sizes, the relative ablation extent was calculated. For the axial histological cuts, a line was drawn between the centers of the insertion sites of the electrodes. Then, both the ablation extent and the total length was measured and put in relation. For the coronary cut slides, the measurement was taken at 0.5 cm of the exposure length to be able to compare to the axial cuts, which were cut roughly at the same length.

## Results

Twelve lesions were produced in the liver of three pigs with a variety of SEE parameters and electrode placement configurations. This was a first large animal study of SEE and therefore we pursued several different goals, such as: testing the effects of SEE parameters, methods and combinations, pre-clinical practice and safety. All the animals survived the procedure without any complication.

The experimental parameters were chosen in such a way as to gain insight into the effects of the various combinations of electrolysis and electroporation on the extent of tissue ablation. Similarly to the evaluation technique in [49,50] we used the extent of the ablated tissue between the electrodes as a measure of the treatment effectiveness. Specifically, we evaluated to what extent the region between electrodes was completely ablated. Table 1 provides details on the treatment parameters in the lesions analyzed in this study and the extent of tissue ablation between the electrodes.

**Table 1:** Details of the treatment parameters.

Experimental parameters				1. Electroporationsequence				Electrolysis		2. Electroporationsequence				Relative Ablation
Fig #	Lesion #	d /cm	e /cm	U /V	p/μs	N	f/Hz	I /mA	t/min	U /V	p /μs	N	f/Hz	ablation/total length
2A, 2B	1	1.5	1	n/a	n/a	n/a	n/a	50	5	n/a	n/a	n/a	n/a	0.40
2C, 2D	2	1.5	1	500	100	16	1	n/a	n/a	n/a	n/a	n/a	n/a	0.00
2E, 2F	3	1.5	1	500	100	8	1	50	5	n/a	n/a	n/a	n/a	0.61
2G, 2H	4	1.5	1	500	100	8	1	50	5	500	100	8	1	0.71
3A	5	2.5	1	n/a	n/a	n/a	n/a	60	10	n/a	n/a	n/a	n/a	0.30
3B	6	2.5	1	3000	100	16	1	n/a	n/a	n/a	n/a	n/a	n/a	0.41
3C	7	2.5	1	3000	100	8	1	60	10	3000	100	8	1	0.77
4A, 4B	8	1.5	1	1000	100	8	1	n/a	n/a	n/a	n/a	n/a	n/a	0.25
4C, 4D, 5, 6	9	1.5	1	1000	100	297	1	n/a	n/a	n/a	n/a	n/a	n/a	0.66
7A–7D, 8, 9	10	1.5	1	1000	100	8	1	75	10	1000	100	8	1	1
7E	11	1.5	1	1000	100	8	1	100	10	n/a	n/a	n/a	n/a	0.97
7F	12	1.5	1	n/a	n/a	n/a	n/a	100	10	1000	100	8	1	0.81

The columns of the table give figure number, lesion number, distance between the electrodes (d; cm) and exposed electrode length (e; cm) for each lesion shown in the figures. This is followed by data for the first electroporation sequence with the parameters voltage (U; V), pulse length (p; μs), amount of pulses applied (N) and frequency (f; Hz). The next column shows the parameters of the electrolysis sequence with the parameters current (I; mA) and time length of delivery (t; min). The next column shows the data for the second electroporation sequence (same parameters as the first sequence). The last column gives the ratio of the extent of tissue ablation between the electrodes and the distance between the electrodes. The line was drawn at about 0.5 cm of exposed length to be able to compare the coronary histological cuts with the axial ones, as the latter was cut at roughly 0.5 cm.

Fig 1 shows photographs from the surgical procedure and ultrasound images taken during the procedure. Ultrasound monitoring was utilized in all the experiments, including electrolysis and multiple pulse electroporation. Fig 1C shows an ultrasound image generated around two Ti needles during a treatment with electrolysis. For comparison, we show in Fig 1D an ultrasound image of a clinically relevant irreversible electroporation procedure performed using four stainless steel needles in a square configuration [44]. The electroporation study in [44] was done by applying a sequence of electroporation pulses, between each adjacent electrodes on each face of the square-like arrangement of electrodes. The sequence consisted of eight 2.5 kV pulses delivered for 100 microseconds at 10 Hz. It is interesting to notice the hyperechoic areas around the electrodes in both electroporation and electrolysis.

**Fig 2:**
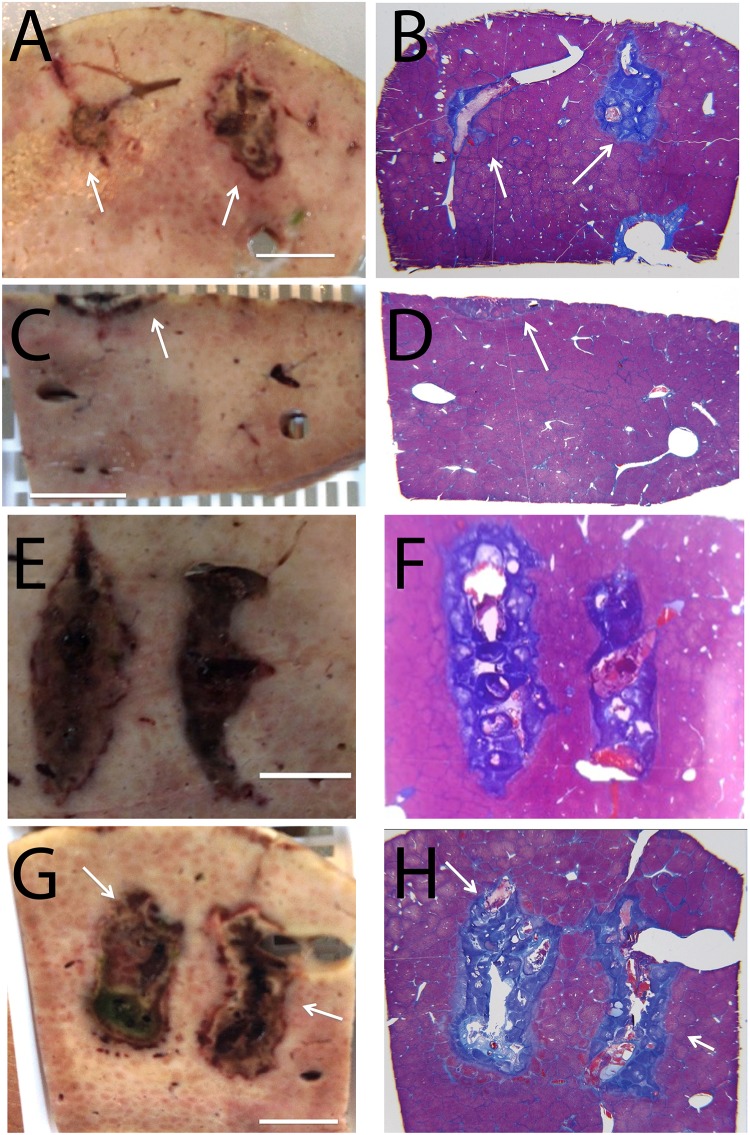
Comparison between extent of tissue ablation from electrolysis, electroporation and combination electroporation and electrolysis. Applied parameters are given in Table 1. (A),(C),(E),(G) Gross macroscopic sections. (B),(D),(F),(H) Trichromatic stained slides. (A) and (B) Electrolysis; 50 mA for 5 minutes. (C) and (D) Electroporation; 500 V, 100 microsecond pulse, 1 Hz, 16 pulses. (E) and (F) Electroporation; 500 V, 100 microsecond pulse, 1 Hz, 8 pulses, followed by electrolysis; 50 mA for 5 minutes. (G) and (H) Electroporation; 500 V, 100 microsecond pulse, 1 Hz, 8 pulses, followed by electrolysis; 50 mA for 5 minutes, followed by electroporation; 500 V, 100 microsecond pulse, 1 Hz, 8 pulses. Scale bar shows 1 cm. Arrows point to lesions.

Fig 2 shows results from a set of experiments, particularly designed to use below-conventional irreversible electroporation voltages, to produce a non-contiguous lesion between the electrodes. This was done to verify conclusions from [49,50] in regards to the synergistic effects of electroporation and electrolysis on tissue ablation. The figure is a comparison between extent of tissue ablation from electrolysis, electroporation and a combination of electroporation and electrolysis. The left column (Fig 2A, 2C, 2E and 2G) shows results from macroscopic sections obtained after flushing the liver with saline and formalin. The dark areas are regions where the blood circulation has ceased and are indicative of regions of cell death [43,44]. The right column (Fig 2B, 2D, 2F and 2H) is for tissues stained with Mason trichromatic stain. The area of cell death from the central core of the lesions (the site of the electrodes) extends to and is demarcated by an outer boundary of pale hepatic cells considered non-viable. The top row (Fig 2A and 2B) shows the appearance of tissue treated by electrolysis with 50 mA for 5 minutes. The lesions have formed around the electrodes, with a gap of intact tissue between them. The minimal width of the unaffected tissue is greater than 1 cm. The second row (Fig 2C and 2D) shows the appearance of tissue treated with 16 electroporation pulses (parameters (a), as described in the text below). No tissue ablation is observed. The ablated tissue on the top of the liver, indicated with an arrow, is a thermal mark made with a bowie to identify the location in which the electrodes were inserted in the tissue. In the samples seen in the second to last row (Fig 2E and 2F) a combination of electroporation and electrolysis was delivered as follows: (a) electroporation; 500 V, 100 microsecond pulse, 1 Hz, 8 pulses, followed by (b) electrolysis; 50 mA for 5 minutes. Here, the ablation ratio is with 0.61 significantly bigger than with electrolysis (0.4) or electroporation (0.0) alone. The samples in the bottom row were treated with a combination of electroporation (a), electrolysis (b) and a second sequence of electroporation with identical electroporation parameters. It is evident that the ablated regions near the electrodes in Fig 2G and 2H are much larger than for the case (a) of electrolysis or case (b) of electroporation alone. While the ablated zone between the electrodes is not continuous, the minimal width of the gap of untreated tissue between the ablated zone is less than 5 mm and much smaller than for electrolysis alone. The relative ablation is 0.71, which is larger than the lesions in Fig 2E and 2F.

**Fig 3:**
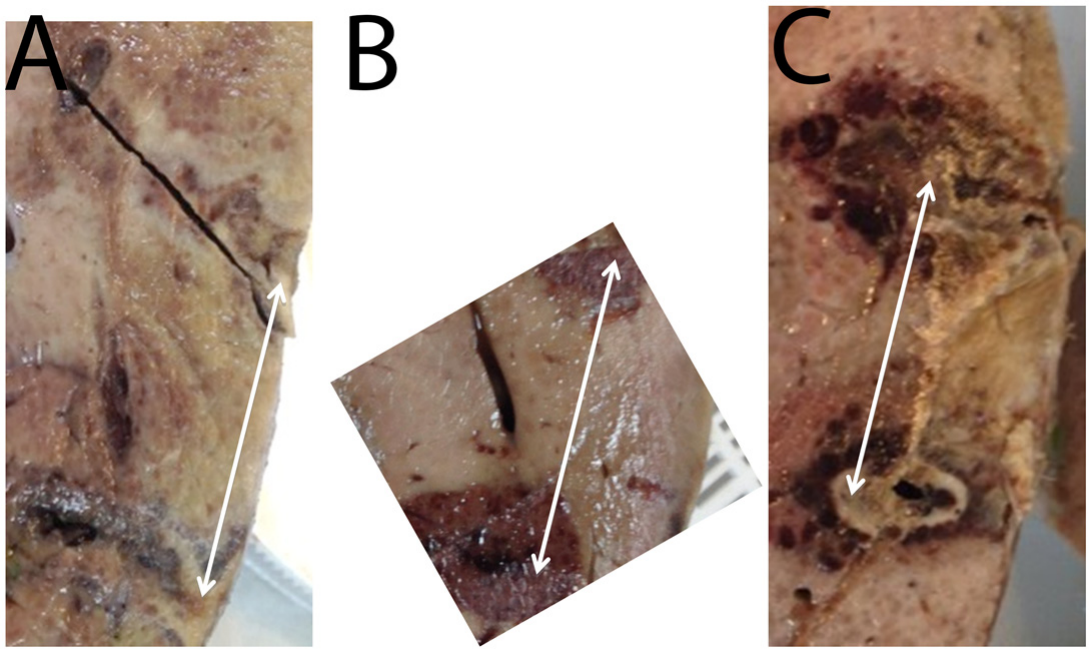
Comparison between extent of tissue ablation from electrolysis, electroporation and the combination electroporation and electrolysis. In this set of experiments, the two Ti electrodes were separated by 2.5 cm. Parameters are given in Table 1. Gross macroscopic section. (A) Electrolysis; 60 mA for 10 minutes. (B) Electroporation; 3000 V, 100 microsecond pulse, 1 Hz, 16 pulses. (C) Electroporation; 3000 V, 100 microsecond pulse, 1 Hz, 8 pulses, followed by electrolysis; 60 mA for 10 minutes, followed by electroporation; 3000 V, 100 microsecond pulse, 1 Hz, 8 pulses.

Fig 3 shows results from another set of experiments, particularly designed to use a larger than conventional irreversible electroporation distance between electrodes to produce a non-contiguous lesion between the electrodes. This was done to verify conclusions from [49,50] in regards to the synergistic effects of electroporation and electrolysis on tissue ablation. Again, the figure compares the extent of tissue ablation from electrolysis, electroporation and a combination of electroporation and electrolysis with two Ti electrodes nominally separated by 2.5 cm (indicated by the arrows). The figure shows results from macroscopic sections obtained after flushing the liver with saline and formalin. The dark areas indicate regions of cell death, as they are the site of ceased blood circulation [44]. The figure compares three types of treatments: A) treated with electrolysis only; B) treated with electroporation only; C) treated with: (a) electroporation, followed by (b) electrolysis, followed by (c) electroporation. The samples were oriented in such a way as to facilitate comparison of the tissues between the electrodes. It is evident that the damage from electrolysis only is negligible and centered around the electrodes. The damage from these high electric field electroporation pulses is more substantial, but the unaffected region between electrodes is of significant size, well over 1 cm. The damage from the combination is greater than from electrolysis or electroporation alone and extends in parts across the entire region between the electrodes.

**Fig 4:**
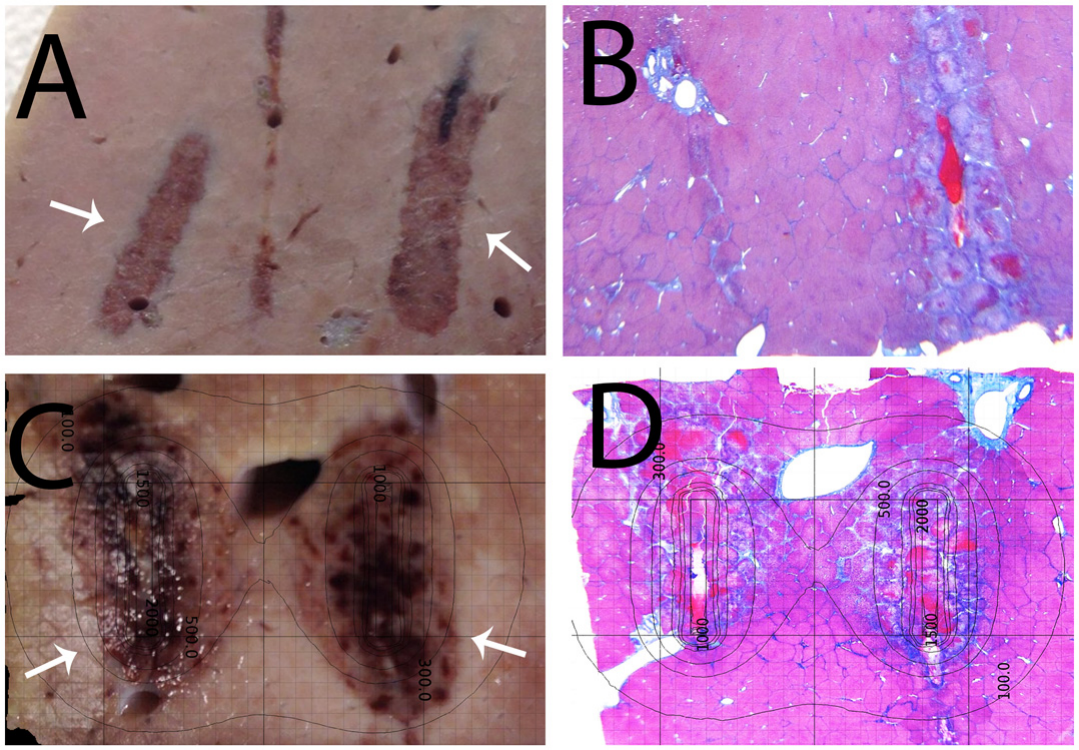
Comparison between extent of tissue ablation by different numbers of electroporation pulses. A detailed list of the parameters can be read in Table 1. (A) and (C) Gross macroscopic sections. (B) and (D) Trichromatic stained slides. Electroporation pulse parameters—1000 V, 100 microseconds, 1 Hz. (A) and (B) 8 pulses. (C) and (D) 297 pulses. Calculated isoelectric field lines are superimposed on the panels in the bottom row. They are also applicable to the top row. In all panels, the anode is the right electrode, while the cathode is on the left.

**Fig 5:**
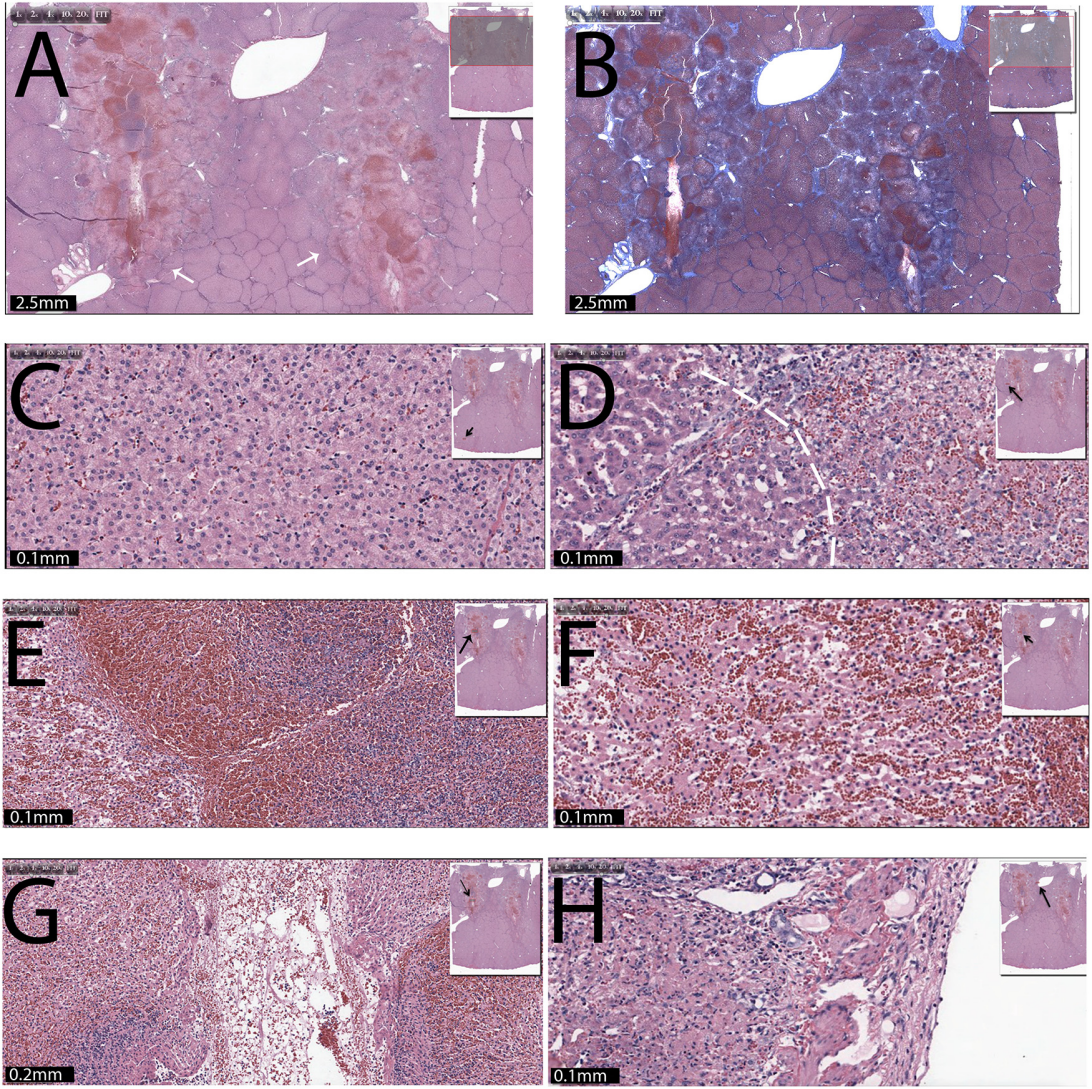
Electroporation with 1000 V, 100 microseconds, 1 Hz, 297 pulses. Applied parameters are given in Table 1. The left electrode is always the anode, while the cathode is on the right side. (A) Magnified H&E and (B) Mason trichromatic stained tissues, focusing on the region between the electrodes. (C) Control. (D) Interface between affected and normal area. (E) The core of the treated region on the left hand side (x10). (F) Magnification (x20) from the core on the left hand side. (G) Left hand side electrode core near a dehydrated region (x10). (H) The left hand side lesion near a large blood vessel. Rectangles and black arrows show the site from which the magnified sample was taken. Scale bars and magnification are given in the figures.

**Fig 6:**
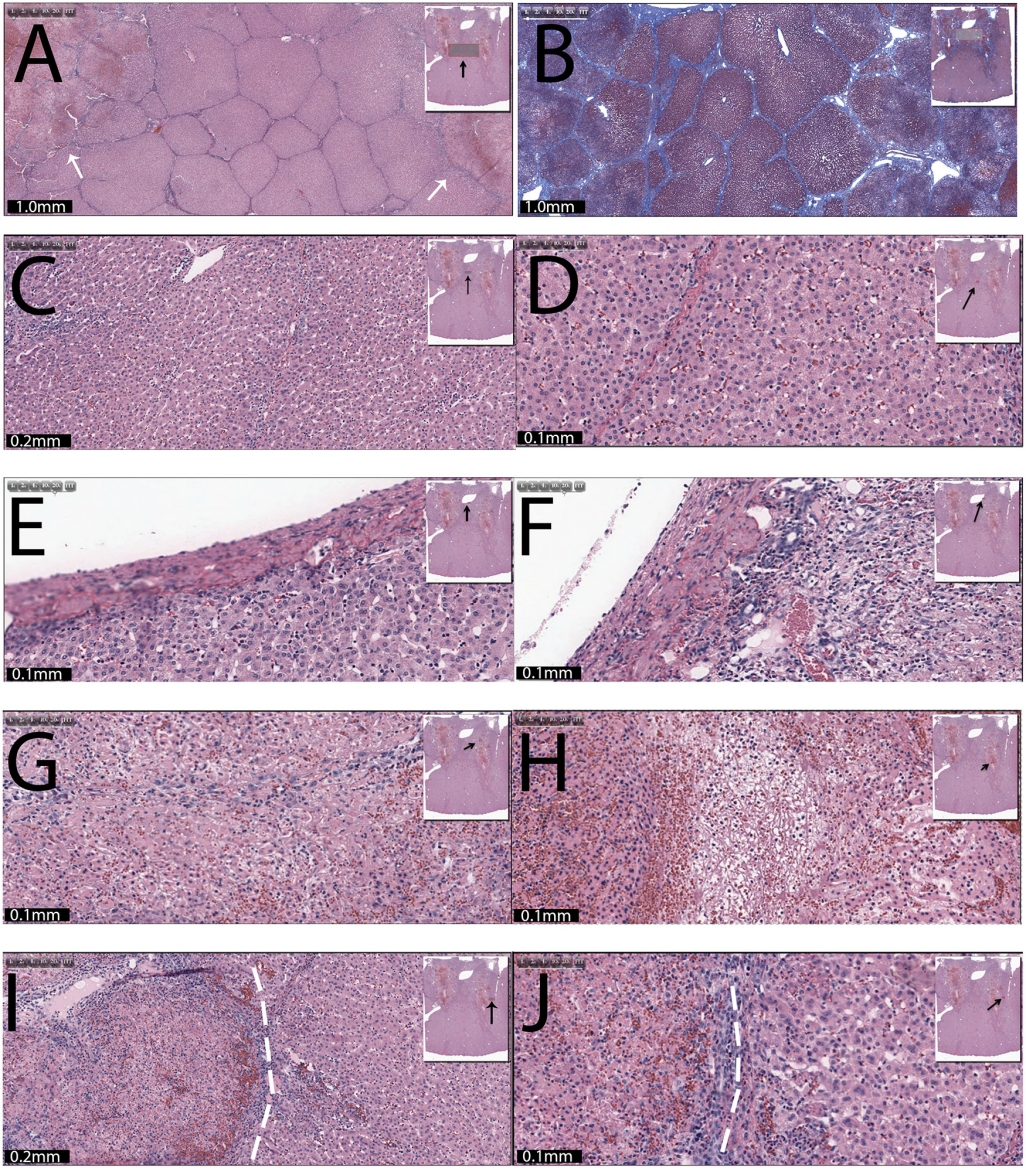
Microscopic details from the 297 pulse treatment. (A) and (B) Low magnification micrographs from different sites of the middle region between electrodes. (C) and (D) 10x and 20x magnification of the tissue in the area midway between the electrodes. (E) and (F) 20x magnification of tissue adjacent to the large blood vessel, with sites in the area midway between the electrodes (E) and tissue near the large blood vessel on the side of the right electrode (F). (G) and (H) Micrographs from sites in the core of the region affected by the right hand side electrode. (I) and (J) Micrographs from sites at the right hand side, outer edge of the right electrode. The sites from which the magnified micrographs were taken are marked with a square in the insert. Scale bars and magnification are given in the figures.

Fig 4 is relevant to the clinical use of the combination electrolysis and electroporation. It is an implementation of the concept introduced in [49] concerning the use of multiple low voltage electric pulses for tissue ablation. The figure compares the ablation caused from 8 typical electrochemotherapy magnitude type pulses (Fig 4A and 4B) to the ablation caused by 297 such pulses (Fig 4C and 4D. The extent of tissue ablation is clearly much larger in the 297 pulse study than in the 8 pulse study. Comsol Multiphysics calculated isoelectric fields produced by the voltages used in this study are superimposed on the images in the bottom panels. It is evident that in the 8 pulses case the ablation extends to electric fields higher than 500 V/cm, which is typical to irreversible electroporation cell damage. In the case of 297 pulses the damage extends to electric fields of 200 V/cm, which are typical to the combination electrolysis electroporation cell damage. Microscopic details from the 297 pulse treatment are shown in Figs 5 and 6.

The top row two panels in Fig 5 are higher magnification of the H&E stain (Fig 5A) and Mason trichromatic stain (Fig 5B) images from Fig 4. Both kinds of staining show a section of the liver with parallel vertical lesions traversing the parenchyma, following the tract of the electrodes. The lesions are characterized as acute tissue necrosis with edema and hemorrhage. The outer boundaries of the tissue injury between the electrodes are pale and swollen. There is a wedge of liver tissue between the lesions (electrode) that appears to be normal and intact (viable). Fig 5C functions as a control and shows the microscopic appearance of the intact liver at a distance from the lesion. The location of the site from which the magnification was taken is shown in the rectangle and marked with an arrow in the insert. This section of the liver has normal and intact cellular details. Fig 5D shows the left margin of the treated zone. There is clear demarcation between the normal hepatocytes on the left compared to the ablated cells on the right (dashed line). The affected cells are swollen and/or contracted with pale cytoplasm and condensed nuclei. Edema and congestion are also noted. Fig 5E shows a 10x magnification of the core of the left lesion. The H&E stained micrograph shows a section of the liver with acute cellular necrosis throughout the field. A more severe effect is present in the left side of the panel, where the cellular architecture is completely destroyed with edema and hemorrhage. The hepatocytes on the right side of the panel are swollen and/or condensed with loss of cytoplasmic detail. There is mild edema and congestion. Fig 5F shows a higher magnification (x20) of Fig 5E. The image is showing necrotic hepatocytes with disrupted sinusoidal pattern. Cells are swollen and/or contracted with dark nuclei. There is congestion/hemorrhage. Fig 5G shows a 10x magnification of an area of the core near a dehydrated region. The section shows an area of extreme treatment related hepato-cellular destruction surrounding the core of the electrode pathway. Notice marked loss of hepatocytes in the core, which is filled with fibrous substance and red blood cells. Fig 5H illustrates an ablated region on the left hand side of a large blood vessel. The section shows a wide field of pale ablated cells adjacent to the vessel. There are small and large pockets containing proteinaceous pink substance. The liver cells on the left side of the panel are compacted. No cellular outline is discernible. Some endothelial cells of the blood vessel may be preserved.

Fig 6 also shows the results of the 297 electroporation pulses case (lesion 9) at other sites in the multiple electroporation treated tissue. Fig 6A and 6B show lower magnification images of the tissue mid-way between electrodes. A large band (or plate) of intact hepatic cells (lobules) are seen between the outer margins of the left and right lesions. Fig 6C and 6D show 10x and 20x magnification micrographs of the tissue in the treated region midway between the electrodes. Well preserved, normal hepatocytes can be seen. Due to the massive damage of the surrounding area, some very mild sinusoidal congestion is visible. Fig 6E shows a magnification of tissue adjacent to a large blood vessel in the area midway between the electrodes. Histologically, the hepatocytes and the endothelial cells of the blood vessel appear intact. In Fig 6F can be seen a section of the ablated region on the right hand side near a large blood vessel. The section shows a wide field of pale ablated cells adjacent to a large blood vessel. The liver cells on the right side of the panel are compacted. No cellular outline is discernible. Some endothelial cell of the blood vessel may be preserved.

The fourth row (Fig 6G and 6H) shows micrographs from the core of the area affected by the right hand side electrode. Fig 6G displays a section of the liver with acute cellular necrosis throughout the field, while Fig 6H shows an area in which the cellular structure is completely disintegrated with marked loss of hepatocytes in the core, filled with fibrous substance and red blood cells. The fifth row (Fig 6I and 6J) illustrates micrographs from sites at the right hand side outer edge of the right side electrode. There is clear demarcation between the normal hepatocytes on the right compared to the ablated cells on the left (dashed lines). The affected cells are swollen and/or contracted with pale cytoplasm and condensed nuclei. Edema and congestion is also visible.

**Fig 7:**
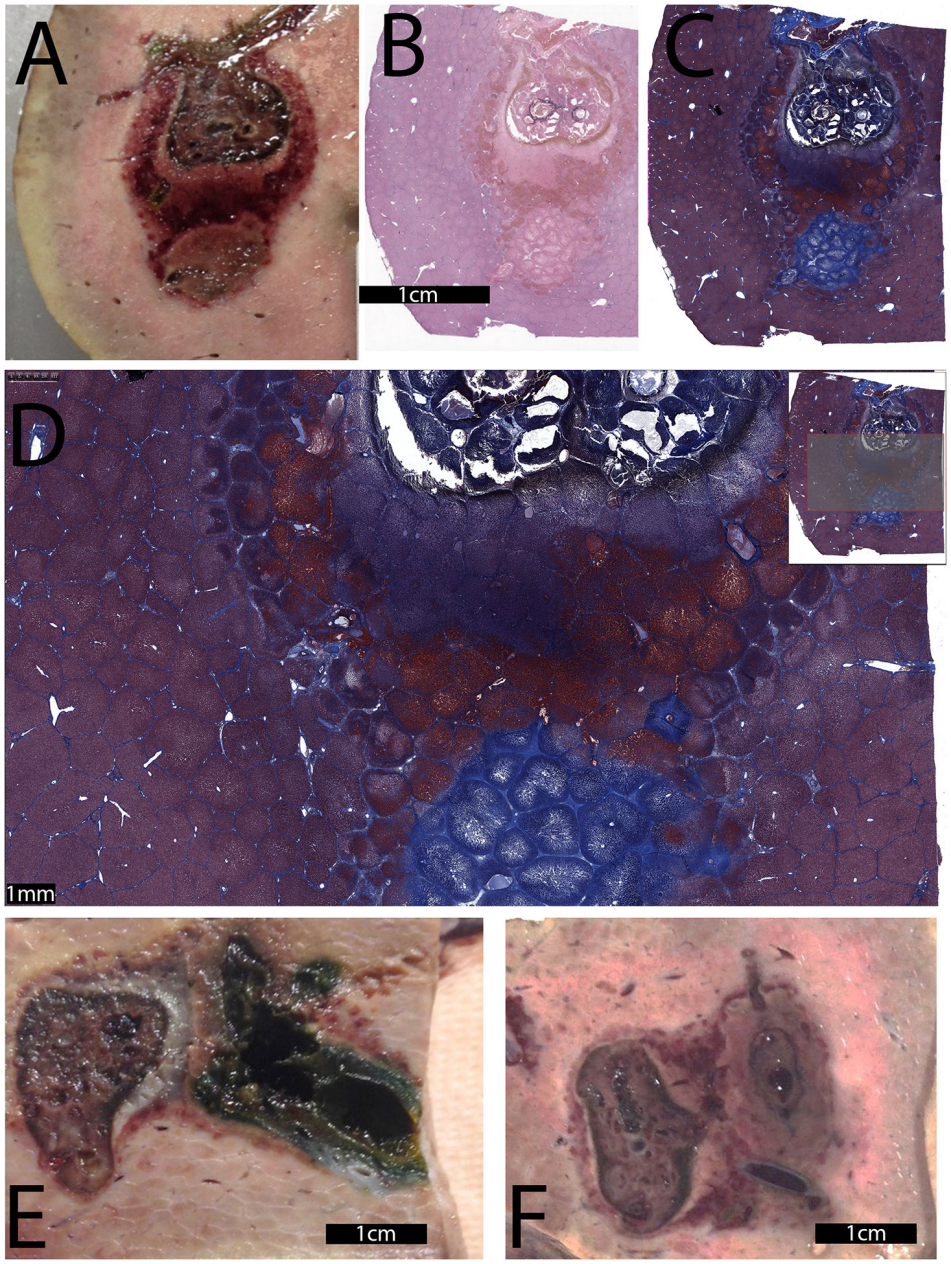
Electroporation followed by electrolysis followed by electroporation. The parameters were: Electroporation—1000 V, 100 microseconds, 1 Hz, 8 pulses followed by electrolysis—10 minutes, 75 mA, followed by electroporation—1000 V, 100 microseconds, 1 Hz, 8 pulses. Used were two Ti electrodes separated by nominal 1.5 cm and exposed length of 1 cm. In this experiment, the top electrode was the anode, while the cathode was placed at the bottom. (A) Macroscopic image. (B) H&E staining. (C) trichromatic stain. (D) Magnified Mason trichromatic stained tissue, focusing on the region between the electrodes. The insert shows a highlighted rectangle representing the region from where the magnification was taken. (E) Electroporation (1000 V, 100 microseconds, 1 Hz, 8 pulses), followed by electrolysis (10 minutes, 100 mA). (F) Electrolysis (10 minutes, 100 mA) followed by electroporation (1000 V, 100 microseconds, 1 Hz, 8 pulses)

Fig 7A–7D shows results from a possible clinical protocol, in which low voltage electroporation pulses are combined with electrolysis. The protocol consists of: typical electrochemical magnitude type electroporation (1000 V, 100 microsecond pulse, 1 Hz, 8 pulses), followed by electrolysis (75 mA for 10 minutes), followed by another typical electrochemical magnitude type electroporation. The treatment was delivered with two Ti needles at a nominal distance of 1.5 cm and an exposed length of 1 cm. The anode is the top electrode and the cathode is the bottom electrode. Fig 7A shows a macroscopic section of tissue, while stained slides can be observed in Fig 7B (H&E staining) and 7C (Mason trichromatic stain). Fig 7D is a magnified micrograph of the region between the electrodes. Here, unlike the case for 297 pulses, the lesion is continuous between the electrodes. The figures of the stained tissues show two distinct adjacent lesions arranged somewhat is a “dumb-bell” shape. The upper lesion is relatively more pronounced and contains severe necrotic acellular debris with charring and edema. The necrotic center is surrounded by a thick circular zone of coagulation necrosis, followed by an outermost zone marked by hemorrhagic liver cell necrosis. The lower lesion is slightly less severe and is characterized by a central region of coagulation necrosis, with the outermost circular thick zone hemorrhagic necrosis merging with the hemorrhagic zone of the upper lesions, without discernible interface or normal liver tissue between the two electrodes induced lesions. Fig 7E and 7F show the results of an experiment with the same combination, but with the permutations electroporation/electrolysis and electrolysis/electroporation. Comparing the lesions shows that all three permutations produced a similar extent of continuous ablation between electrodes, which illustrates that the exact sequence of the permutations of electrolysis and electroporation does not affect the results. On the contrary, the delivery of 297 electroporation pulses of the kind did not produce an uninterupted lesion.

**Fig 8:**
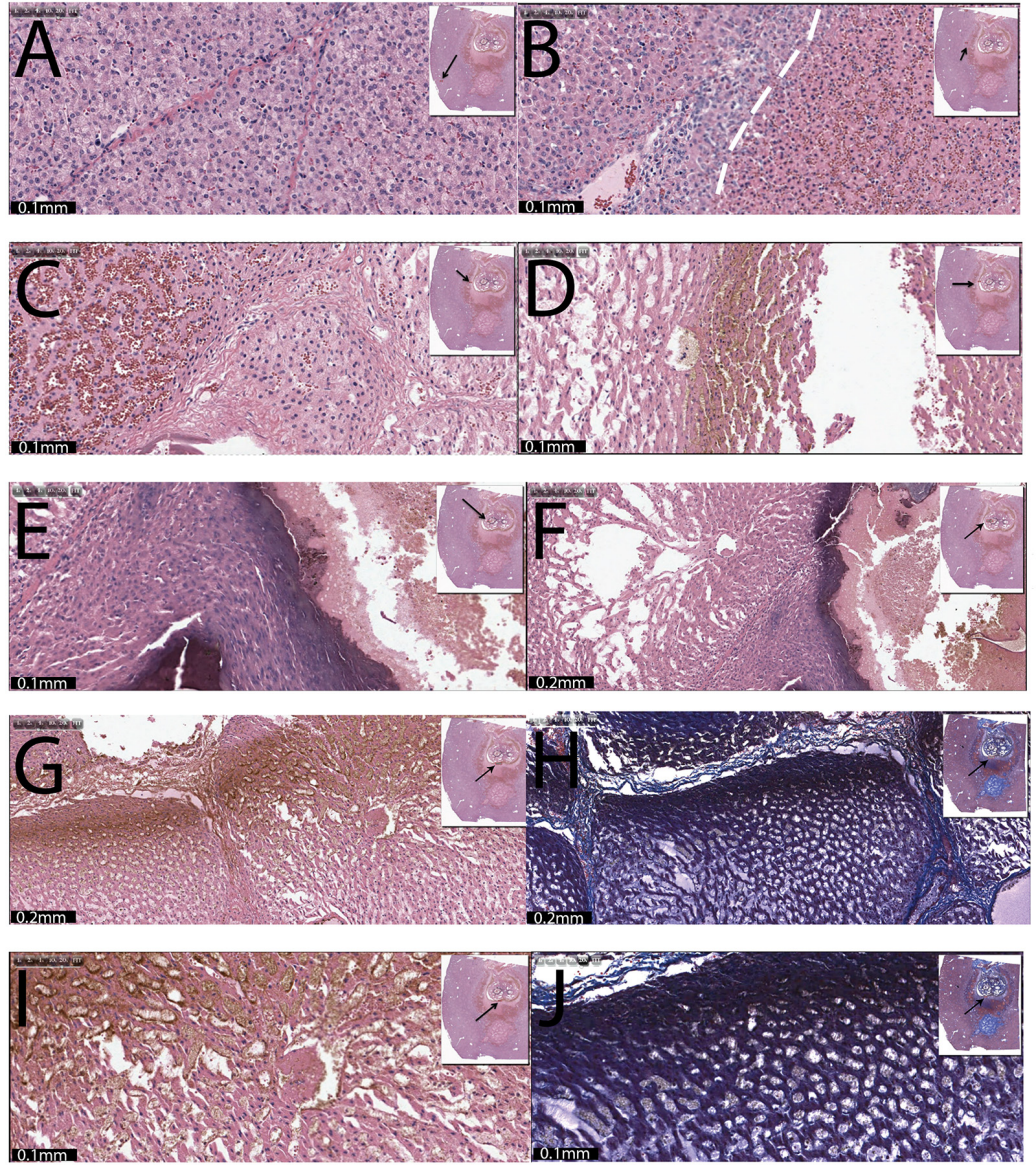
Electroporation followed by electrolysis followed by electroporation. The lesions shown are in higher magnification than illustrated in Fig 7, but are from the same treatment taken at different sites. (A) Control. (B) This section of the liver stained with H&E contains normal liver cells with no evidence of treatment related injury; outer edge of lesion at the anode side. (C) and (D) Micrographs towards the core of the anode treated region. (E) and (F) Micrographs at the anode core with 20x (E) and 10x (F) magnification. (G)—(J) Sites at the margin of the anode affected region towards the cathode, with (G) and (H) in 10x and (I) and (J) in 20x magnification. Tissues were stained in H&E ((G), (I)) and Mason trichromatic stain ((H), (J)).

The lesion in Fig 8 is in higher magnification than illustrated in Fig 7, but shows the same treatment taken at different sites. The sites from which the images are taken are marked in the insert with a highlighted rectangle and a black arrow. The magnification and scale bar are given in the figures. The control image in Fig 8A shows a section of the liver stained with H&E, which contains normal liver cells with no evidence of treatment related injury. Fig 8B shows the edge of the lesion at the anode side. This section of the liver stained with H&E shows acutely necrotic liver cells at the margin of the lesion. A more profound necrosis can be seen on the right side where the cellular architecture is affected and has hemorrhage (dashed line). A relatively narrow region of delineation occurs between the affected tissue on the right and the normal tissue on the left. It is seen as a pale zone that is less affected and is mixed with cells that appear normal. The cells on the very left appear normal. Fig 8C displays an area towards the core of the anode treated region. This section of the liver is stained with H&E and shows severe coagulation with congestion/hemorrhage. This is followed by another site towards the core of the treated region near the anode (Fig 8D). This section of the liver, stained with H&E, has massive tissue necrosis with complete architectural disruption. The most extreme right edge is fragmented and probably dehydrated (shrinkage), followed by lobules that have thin strips of liver cells separated by clear spaces (edema). There is also coagulation necrosis and hemorrhage. The third row (Fig 8E and 8F) shows the appearance of the tissue at the core, near the anode at 20x (Fig 8E) and 10x (Fig 8F) magnification. The H&E stained section shows a markedly necrotic center containing acellular tissue debris bordered by a dark band of coagulated tissue with loss of cellular details. This is followed by a zone of dehydrated or condensed hepatocytes with edema. All cells in this region are necrotic. Fourth and fifth row (Fig 8G–8J) show micrographs of the edge of the anode affected region towards the cathode. The sections are stained with H&E and trichromatic stains and captured at 10x (Fig 8G and 8H) and 20x (Fig 8I and 8J) magnification. Sections have severe necrosis of the liver cells in each of the lobules. The cells are completely effaced. Sinusoidal edema is also present.

**Fig 9:**
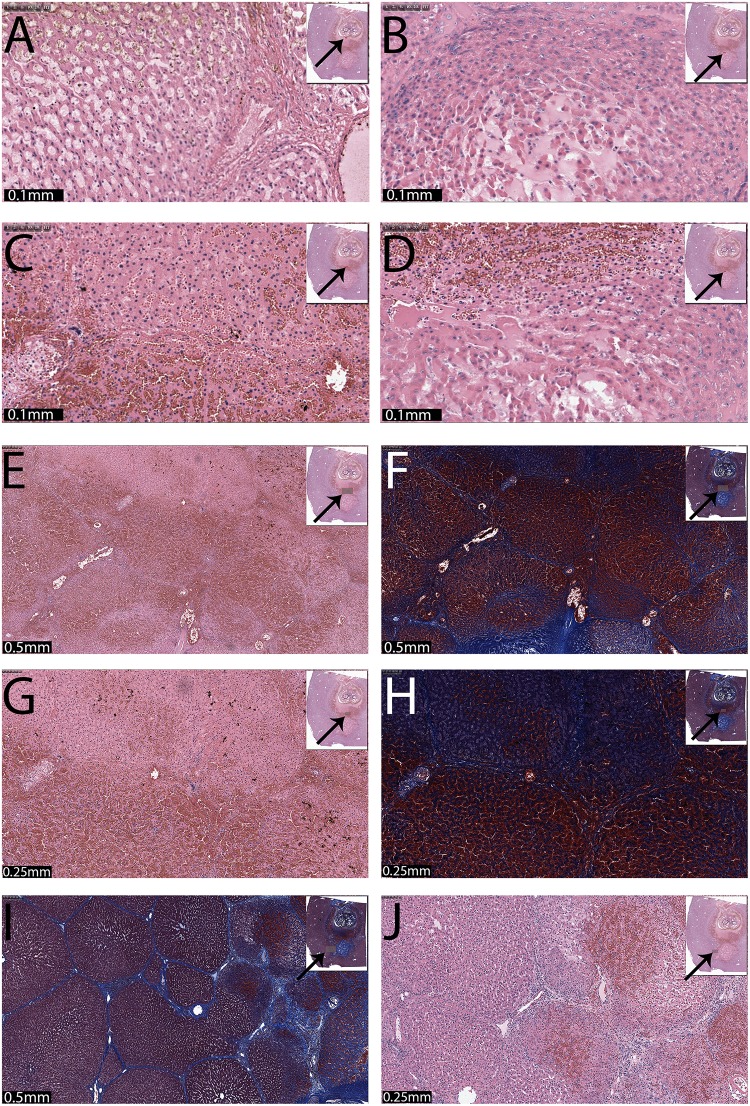
Higher magnification micrographs of the treatment in Fig 7. (A) and (B) Midway between the anode and cathode. (C) and (D) Midway between anode and cathode towards the cathode (x10). (E) The core of the ablated region near the cathode in 10x magnification with H&E (F) and Mason trichromatic stain. The core of the ablated region near the cathode with 20x magnification and H&E staining (G) and with Mason trichromatic stain (H). (I) and (J) Micrographs at the outer margin of the cathodic lesion: (I) 10x magnification and trichromatic stain, (J) 20x magnification and H&E stain.

Fig 9 shows higher magnification micrographs of the treatment in Fig 7, but samples were taken at different sites which are marked in the insert with a highlighted rectangle and an arrow. The magnification and scale bar are given in the panels. Fig 9A shows a site midway between the anode and cathode, towards the anode in H&E staining. The liver cells in the lobules are affected by acute necrosis and have either swollen and/or condensed cytoplasm with dark attenuated nuclei. Some sinusoidal edema is also present. Fig 9B shows a site midway between the anode and cathode towards the cathode. This H&E section shows the necrotic hepatic region gradually transiting into a zone of hemorrhagic necrosis below, towards the cathode. The liver cells have inconspicuous cell boundaries and their nuclei are attenuated. The transition between the tissue ablation modality near the anode to that near the cathode is continued in the panels of the second row (Fig 9C and 9D). Fig 9C shows an H&E stained section which has hemorrhagic necrosis on the top and gradually transitions into more compact and necrotic lobules at the bottom. Further towards the cathode (Fig 9D) is a H&E stained section that has hemorrhagic necrosis on the top with abrupt transiting into cellular necrosis and edema at the bottom. Third and fourth rows (Fig 9E–9H) show the core of the ablated region near the cathode at 10x and 20x magnification, with H&E staining (Fig 9E and 9G) and with Mason trichromatic stain (Fig 9F and 9H). The sections expose acute necrosis of the liver cells in individual lobules. Notice the liver cells are becoming desiccated and individualized with sinusoidal edema. There is a substantial difference between the appearance of the tissue at the core of the lesion near the anode and near the cathode. The anode inflicted massive necrosis that spread to an extensive area of the liver surrounding the core. The core was reduced into mangled acellular tissue debris and edema. The immediately surrounding zone had a thick plate of coagulated liver cells followed by a wide zone of hemorrhagic necrosis. The outermost zone was delineated by acute necrosis and cell swelling. In comparison, the cathode at the bottom inflicted a less severe but complete necrosis of the liver cells confined to narrow proximity. The affected fields somewhat maintained the lobular pattern but the hepatocytes sustained cell death. Fifth row (Fig 9I and 9J) shows micrographs at the outer margin of the cathodic lesion. Fig 9I (10x magnification) shows the lesion with trichromatic stain, and Fig 9J (20x magnification) was stained with H&E. Both stained slides show a field of hemorrhagic necrosis (top left) interfacing with a field of acutely necrotic liver cells in the bottom half of the section.

## Discussion

The primary goal of this study was to expand on our earlier small animal study [49,50] and examine tissue ablation protocols that combine electrolysis and electroporation in a configuration and with parameters that are relevant to clinical applications. Since our earlier work on tissue ablation with non-thermal irreversible electroporation (NTIRE) [44, 51], medical imaging has become standard in clinical use of NTIRE. We therefore considered using ultrasound imaging in our study as well. While performing the electrolysis studies under ultrasound, we have observed in all the experiments the bright hyperechoic appearance in the region adjacent and between the electrodes. An example is shown in Fig 1C. Since electrolysis is known to produce gases near the electrodes, the most likely explanation is that these bright areas are the reflection of ultrasound waves from the gas tissue interface. This led us to re-examine ultrasound images from our earlier irreversible electroporation studies [44,51]. Fig 1D illustrates a typical site of NTIRE treated tissue immediately after the procedure. Comparing the panels in the bottom row (Fig 1C and 1D), it is now obvious that the bright spots near the electrodes in the NTIRE procedure are due to gases produced by electrolysis during the electroporation pulses. This has important clinical value. It is common to experience loud and sudden explosion-like sounds during NTIRE protocols. The observed bright areas now explain the mechanism: These sounds are most likely the result of an electric discharge across the electrolysis generated layer of gas around the electrodes. Electroporation employs voltages as high as 3000 V. When the field across the layer is higher than about 3000 V/cm, the gases ionize and an electric discharge akin to lightning occurs. For a voltage of 3000 V, a 100 micron thick layer of gas will be sufficient to cause a discharge. This discharge generates high pressure waves that could be detrimental to the treated organ, in particular if it is encapsulated such as the brain, bone or the prostate. Monitoring the formation of bright spots at the electrodes during electroporation could be used to avoid the electric discharge.

Fig 2 shows a set of four experiments that confirm the synergistic effect of the combination of electrolysis and electroporation. The top row (lesion 1) shows the extent of tissue ablation in a pure electrolytic procedure. The current delivered and the time of delivery are substantially lower and shorter, respectively, than conventional protocols for tissue ablation by electrolysis. Indeed the ablation occurs primarily near the electrodes, with an ablation ratio of 0.4. The second row (lesion 2) shows the effect of electroporation with parameters which are typical to reversible electroporation. Indeed, the panels show that the applied voltage has no effect on the tissue. The third row shows the results of the combination of electroporation and electrolysis with the same parameters used for lesion 1 and 2. The ablated area has significantly increased, as can be seen in Fig 2E and 2F. The last row results (lesion 4) are consistent with the synergistic effect of the combination of two sequences of electroporation and electrolysis, also reported in [50]. The extent of tissue ablation is substantially larger than in the other tissue treatment panels, with an ablation ratio of 0.71. As discussed in the introduction, the mechanism probably involves using conventional electrolysis as the central tissue ablation modality and additionally using electroporation pulses to permeabilize the cell membrane to make the cell more susceptible to lower amounts of products of electrolysis [50]. This figure suggests some possible clinical implications. It seems that 500 V, which is usually used in reversible electroporation, may be sufficient to produce an approximately 2 cm ablation lesion, when delivered in combination with electrolysis, from two Ti electrodes separated by 1 cm.

Another set of three experiments that confirm the synergistic effect of electrolysis and electroporation are shown in Fig 3. In this case we increased the distance between the electrodes to 2.5 cm. Fig 3A shows the effect of delivering a low non-clinical dose of electrolysis, which shows that little damage was produced, just adjacent to the electrodes. The majority of the tissue in the 2.5 cm gap between the electrodes is unaffected. Fig 3B displays tissue treated with electroporation with a voltage that is typical for non-thermal irreversible electroporation (NTIRE) protocols. However, the distance between the electrodes is larger than used in clinical practice. Indeed, the damage occurs primarily around the electrodes. The minimal width of the area unaffected by the treatment is about 1.5 cm. The survival of tissue between electrodes is of clinical concern in NTIRE. Fig 3C shows the outcome of a treatment that combines the electrolysis and the electroporation in the previous two panels. It is evident that although the tissue ablation has a dumb-bell shape, the ablation zone from the two electrodes touch. The appearance of the ablated tissue is different near the two electrodes. This phenomenon is consistent with the mechanisms of electrolytic ablation and was discussed in [49,50]. While much work remains to be done to optimize the protocols that combine electrolysis and electroporation, the two studies in Figs 2 and 3 illustrate the potential. The combination could generate tissue ablation with lower voltages or with larger distances between electrodes.

Fig 4 illustrates an attempt to implement the multiple low voltage electroporation pulse concept described in [49] for tissue ablation in a clinical relevant configuration. The study described in [49] shows that multiple low electric field pulses, of the kind used in reversible type electroporation, can cause tissue ablation. The mechanism involves the penetration of the electrolytic products generated during the delivery of the electroporation pulses into the reversible permeabilized cell membrane. Fig 4 evaluates the effect of the number of low electric field pulses on the extent of tissue ablation. The top row (lesion 8) shows the extent of tissue ablation when 8 pulses are applied. This is the type of protocol that would be used in reversible electroporation treatment of tissue. The results show that the damage is minimal, just adjacent to the electrode needles. Indeed, this is what is hoped for in reversible electroporation. The second row (lesion 9) shows the extent of tissue ablation when 297 pulses are applied. The frequency of 1 Hz was chosen because it is used in conventional NTIRE. However, voltages between 2 kV and 3 kV would be used for this geometrical configuration in conventional NTIRE. The number of pulses used in conventional NTIRE, between two electrodes, is substantially smaller. Therefore, the protocol tested here employs voltages that are a factor of 2 and 3 smaller than in NTIRE and employs numbers of pulses that are a factor of 3 to 6 larger than in conventional NTIRE. Fig 4C and 4D (lesion 10) point out that the extent of tissue ablation by the 297 pulse protocol is substantially larger than that for the 8 pulse protocol. This is consistent with the findings of [49] and demonstrates that the combination of low electric fields with pulse generated electrolytic products can be used for tissue ablation in a clinical relevant setting. The overlaid calculated isoelectric field lines show that while the extent of tissue ablation with 8 pulses is encompassed by isoelectric fields of about 1000 V/cm and higher, the extent of tissue ablation with 297 pulses seems to extend to isoelectric field lines of 300 V/cm and even 200 V/cm. The electric fields of 200 V/cm and 300 V/cm are in the domain of reversible electroporation, which cells survive in the absence of electrolysis. The ablation shown in Fig 4 has a dumb-bell shape, with what appears to be intact tissue in the middle area between the electrodes. The electric fields have a similar dumb-bell shape. Fig 5A and 5B are higher magnification of the H&E stained (Fig 4A) and Mason trichromatic stain (Fig 4B) images from Fig 4. They focus on the region between the electrodes. Both kinds of staining show a section of the liver with parallel vertical lesions traversing the parenchyma, following the tract of the electrodes. The lesions are characterized as acute tissue necrosis with edema and hemorrhage. The outer boundaries of the tissue injury between the electrodes are pale and swollen. There is a wedge of liver tissue between the lesions (electrode) that appear to be normal and intact (viable). Perhaps the most interesting observation from bottom row in Fig 4 (Fig 4C and 4D) and top row of Fig 5 (Fig 5A and 5B) is that the macroscopic margin of cell death, while convoluted, follows quite precisely the isoelectric field lines, showing that the mechanism is related to the process of electroporation. It is also interesting to notice that the isoelectric field lines that are followed correspond to parameters for reversible electroporation. This supports the idea that the mechanism of damage in this protocol involves the synergistic combination of electrolysis with electroporation. It should be noted that the length of this procedure was about 8 minutes with the BTX. It is also interesting to notice that the extent of tissue ablation with the multiple electroporation pulse protocol is somewhat similar to that obtained with a combination of 5 minutes electrolysis and 16 500 V electroporation pulses. This aspect will be discussed later in the context of Fig 7.

Next we will follow, with high magnification, a path from the outer edge of the left electrode (anode) to the outer edge of the right electrode (cathode). Fig 5C shows a control sample from the same liver for comparison with treated tissue. It will become evident, as we go through the micrographs that the patterns of cell death change from point to point in the treated region. This was also observed in [49]. Fig 5D illustrates the appearance of the treated tissue at the left hand side margin. There seems to be a relatively sharp transition between the normal hepatocytes on the left and the swollen and/or contracted cells with pale cytoplasm and condensed nuclei on the right. This sharp transition is a feature of cells affected by electroporation and was also observed in [43]. Edema and congestion are also noted in the treated region, which is a known feature of cell ablation by electroporation [43]. It is important to notice that the edema and congestion extend to the margin of the treated lesion. This is what gives the macroscopic gross sections the dark appearance and suggests that, indeed, the dark margin in the macroscopic gross sections can be taken to approximately represent the margin of the lesion.

Fig 5E shows a 10x magnification at a site in the core of the left hand side lesion. The H&E stained micrograph shows a section of the liver with acute cellular necrosis throughout the field. It should be noted that the lobular structure is nevertheless retained. Another interesting observation is the appearance of the ablated cells which is different in the right hand side from the left hand side of the panel. A higher magnification from the same site is shown in Fig 5F. It is evident that the damage to the cells is major. The image is showing necrotic hepatocytes with disrupted sinusoidal pattern. Cells are swollen and/or contracted with dark nuclei, and there is congestion/hemorrhage. This region has experienced complete cell ablation.

The fourth row (Fig 5G and 5H) shows that adjacent to the electrodes, the core region seems to be completely devoid of any cellular structure with marked loss of hepatocytes in the core, which is filled with fibrous substance and red blood cells. This may be related to electro-osmotic water flow from the anode to the cathode, which is typical to processes of electrolysis. Fig 5G in particular is interesting because it focuses on the interface between ablated tissue and a large blood vessel. The section shows a wide field of pale ablated cells with small and large pockets containing proteinaceous pink substance. The liver cells on the left side of the panel are compacted. No cellular outline is discernible. However, it appears that the morphological structure of the large blood vessel is intact and it has remained patent, i.e. not occluded. The preservation of large blood vessels architecture by both electrolysis [23,24] and electroporation [45] separately, are considered a major advantage of these ablation modalities. This is what allows their use to treat tumors near large blood vessels. It now seems that the combination of electroporation and electrolysis also maintains the large blood vessels functionality. Obviously, this is only a 24 hours survival study and longer studies are required to further verify this important aspect of the combination of electroporation with electrolysis as an ablation procedure.

Fig 6A and 6B show a low magnification of the area midway between the electrodes. The liver in this area is normal and unaffected by the treatment, as evident from the higher magnification micrographs in Fig 6C, 6D and 6E. The third row shows the appearance of tissue near the large blood vessel mid-way between the electrodes (Fig 6E) and near the right electrode (Fig 6F). In contrast with the normal appearance of the tissue adjacent near the blood vessel, midway between the electrodes, the cells near the right electrodes are completely ablated. Nevertheless, as on the left side of the blood vessel, here also the structural integrity of the blood vessel has been apparently retained.

The forth row (Fig 6G and 6H) shows micrographs from the core of the area affected by the right hand side electrode. Fig 6G shows a section of the liver with acute cellular necrosis throughout the field. Fig 6H, on the other hand, illustrates an area in which the cellular structure is completely disintegrated with marked loss of hepatocytes in the core, which is filled with fibrous substance and red blood cells. The ablated tissue appearance near the left electrode is comparable to that near the right electrode. Similar to the left hand side margin of the lesion here also, in slides close to the edge of the lesion, there is clear demarcation between the normal hepatocytes on the right compared to the ablated cells on the left. Edema and congestion are also noted on the left hand side margin.

The study in Figs 3–6 show that multiple low voltage electroporation pulses create a much larger ablated zone. The ablated zone seems to be congruent with the isoelectric field lines of the parameters of reversible electroporation, which suggest that the mechanism of damage is the synergy of electroporation and electrolysis. The appearance of the ablated cells changes throughout the lesion. This seems to be a feature of the combination, because the mechanisms of damage are different throughout the treated zone. We believe that there are at least five mechanisms: dominant anodic ablation, dominant cathodic ablation, combination reversible electroporation and anodic compounds, combination reversible electroporation and cathodic compounds and a small region of irreversible electroporation. This suggests that using the combination electroporation and electrolysis requires a good understanding of the biophysics of the process and careful treatment planning. While the ablation is not continuous, it is obvious that these type of protocols combining electrolysis with electroporation through the delivery of multiple low voltage pulses could find clinical application. A major advantage over conventional NTIRE is the use of much lower voltages.

Figs 7A–7D, 8 and 9 show a different possible clinical protocol that employs the combination of electrolysis and electroporation in a mode explored in reference [49]. Specifically, we used conventional electrolysis as the central tissue ablation modality and added electroporation pulses to permeabilize the cell membrane, making the cell more susceptible to lower amounts of products of electrolysis. We tested the concept with a protocol that may become clinical. It combines low voltage electroporation pulses with short periods of electrolysis. The protocol employs voltages that are lower than in conventional NTIRE and the time of electrolysis is much shorter than in conventional electrolysis. Fig 7A–7D shows macroscopic images of the treated tissue. The lesion is made of two distinct adjacent lesions, arranged somewhat is a “dumb-bell” shape, similar to the shape obtained with the multiple pulse protocol. However, unlike the multiple pulse protocol, in this case the outer margins of the necrotic zones mingle with each other and the ablation is continuous, forming an oval shape. The macroscopic images show a variety of cell ablation modalities, which, as with the multiple electroporation pulses, can be attributed to the different mechanisms of cell death. For instance, the upper lesion around the anode is relatively more pronounced and contains severe necrotic acellular debris with charring and edema. The necrotic center is surrounded by a thick circular zone of coagulation necrosis followed by an outermost zone marked by hemorrhagic liver cell necrosis. The lower lesion is slightly less severe and is characterized by a central region of coagulation necrosis, with the outermost circular thick zone hemorrhagic necrosis merging with the hemorrhagic zone of the upper lesions with no discernible interface or normal liver tissue between the two electrodes induced lesions.

The lesions 11 and 12 shown in Fig 7E and 7F serve as a comparison of different permutations of the combination. Lesion 11 shows the effects of electroporation followed by electrolysis, while lesion 12 illustrates the affected tissue after electrolysis followed by electroporation. All three permutations produced a similar extent of ablation that was continuous between the electrodes. This shows that the exact sequence of the permutations of electrolysis and electroporation does not affect the results.

Figs 8 and 9 show higher magnification of the tissue ablation, in a progression from the outer edge of the ablated tissue near the anode to the outer edge of the ablated tissue near the cathode. This should facilitate a better understanding of the different mechanisms of action during the combination tissue ablation process.

In Fig 8A we see a section of the liver stained with H&E which contains normal liver cells with no evidence of treatment related injury. Fig 8B illustrates the edge of the lesion at the anode side. The appearance is rather similar to that in Fig 5D. Here there is also profound necrosis on the right hand side of the panel, with substantial hemorrhage, separated by a relatively narrow region of delineation between the affected tissue on the right and the normal tissue on the left. The cells on the very left appear normal. As in the multiple pulses case and in NTIRE, the interface between ablated and living cells is very narrow. The sharp delineation is considered an advantage of NTIRE and it apparently occurs also in this combination of electrolysis and electroporation. A possible explanation is that the opening of the cell membrane by electroporation is required for cell death. Electroporation is a relatively binary process, and this may explain the narrow range of transition. The hemorrhage is also typical to electroporation and, like in the previous study, supports the use of macroscopic gross sectioning of tissue to evaluate the extent of ablated tissue.

The second and third rows (Fig 8C–8F) show areas towards the core of the anode treated lesion. The damage here is much more massive than in the multiple electroporation pulses case. There is severe coagulation, massive tissue necrosis, as well as fragmented and dehydrated strips of liver tissue. In this case there was much more electrolysis than in the multiple electroporation pulse case, and this more massive mechanism of damage can be attributed to the electrolytic ablation. The dark band of coagulated tissue is probably due to the chlorine species that form at the anode in a chemical reaction. The entire core region seems to be severely dehydrated. This can be explained by the electro-osmotic flow of water from the anode to the cathode. In fact the low magnification images in Fig 7 support this model. It is possible to observe a rim of condensed red blood cells around the anode affected region, concentrating in the region between the anode and cathode. A possible explanation is that this could be due to the flow of water from the anode to the cathode has carried red blood cells, which were then deposited at the interface of the cathode affected region. The higher magnification micrographs in Fig 8 rows four and five (Fig 8G–8J) and Fig 9 rows one and two (Fig 9A–9C) show that indeed, the damage is much less severe than around the anode and there is a high conglomeration of red blood cells with hemorrhagic necrosis. The appearance of the necrotic core near the cathode is substantially different from that near the anode. In comparison to the anode, the cathode at the bottom inflicted a less severe but complete necrosis of the liver cells. It is interesting to notice that the lobular pattern is retained. The margin of the treated region near the cathode is shown in Fig 9I and 9J. Similarly to the margin of the lesion near the anode and the margins of the lesion near the anode and cathode with the multiple electroporation pulse protocol, the transition region between dead and live cells is narrow and characterized by hemorrhagic necrosis.

Evidently, in this protocol also, the mechanisms of damage vary as a function of the proximity to the anode or cathode. This phenomenon was also observed by others working in the field of electrolytic ablation [23,24]. and us There is a clinical significance to that. It means that in protocols that combine electrolysis and electroporation, it is important to place the cathode and anode in an optimal configuration in regards to the nature of the treated tissue. For example, it tentatively appears that it would be preferential to place the cathode near a sensitive tissue that is to be spared, such as a blood vessel.

## Conclusion

Using a large animal model, we have confirmed the findings in [49,50] that a synergistic combination of electrolysis and electroporation can produce more effective ablation than either electrolysis or electroporation separately. Furthermore, this combination lends itself to the design of clinical protocols that employ lower voltages than NTIRE and shorter times than electrolysis. Obviously, this is only a first large animal study of this combination tissue ablation modality. Substantial research remains to be done to optimize the concept for clinical use.
